# Phenyl *N*-(*p*-tol­yl)carbamate

**DOI:** 10.1107/S1600536809022600

**Published:** 2009-06-17

**Authors:** Shu-Xin Bao, Zheng Fang, Yong-Lu Wang, Ping Wei

**Affiliations:** a School of Pharmaceutical Sciences, Nanjing University of Technology, Xinmofan Road No. 5 Nanjing, Nanjing 210009, People’s Republic of China; b College of Life Sciences and Pharmaceutical Engineering, Nanjing University of Technology, Nanjing 210009, People’s Republic of China

## Abstract

The asymmetric unit of the title compound, C_14_H_13_NO_2_, contains two crystallographically independent mol­ecules, in which the aromatic rings are oriented at dihedral angles of 59.01 (3) and 56.98 (3)°. In the crystal structure, inter­molecular N—H⋯O hydrogen bonds link the mol­ecules into chains.

## Related literature

For bond-length data, see: Allen *et al.* (1987 ▶).
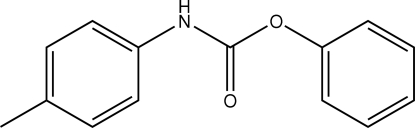


## Experimental

### 

#### Crystal data

C_14_H_13_NO_2_
*M* *_r_* = 227.25
Triclinic, 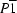

*a* = 8.7790 (18) Å
*b* = 9.7470 (19) Å
*c* = 15.121 (3) Å
α = 87.30 (3)°
β = 77.07 (3)°
γ = 75.00 (3)°
*V* = 1218.0 (5) Å^3^
*Z* = 4
Mo *K*α radiation
μ = 0.08 mm^−1^
*T* = 294 K
0.30 × 0.20 × 0.10 mm

#### Data collection

Enraf–Nonius CAD-4 diffractometer
Absorption correction: ψ scan (North *et al.*, 1968 ▶) *T*_min_ = 0.975, *T*_max_ = 0.992
4736 measured reflections
4421 independent reflections
2781 reflections with *I* > 2σ(*I*)
*R*_int_ = 0.027
3 standard reflections frequency: 120 min intensity decay: 1%

#### Refinement

*R*[*F* ^2^ > 2σ(*F* ^2^)] = 0.054
*wR*(*F* ^2^) = 0.172
*S* = 1.01
4421 reflections
308 parameters
H-atom parameters constrained
Δρ_max_ = 0.22 e Å^−3^
Δρ_min_ = −0.13 e Å^−3^

### 

Data collection: *CAD-4 Software* (Enraf–Nonius, 1989 ▶); cell refinement: *CAD-4 Software*; data reduction: *XCAD4* (Harms & Wocadlo, 1995 ▶); program(s) used to solve structure: *SHELXS97* (Sheldrick, 2008 ▶); program(s) used to refine structure: *SHELXL97* (Sheldrick, 2008 ▶); molecular graphics: *ORTEP-3 for Windows* (Farrugia, 1997 ▶) and *PLATON* (Spek, 2009 ▶); software used to prepare material for publication: *SHELXL97* and *PLATON*.

## Supplementary Material

Crystal structure: contains datablocks global, I. DOI: 10.1107/S1600536809022600/hk2710sup1.cif

Structure factors: contains datablocks I. DOI: 10.1107/S1600536809022600/hk2710Isup2.hkl

Additional supplementary materials:  crystallographic information; 3D view; checkCIF report

## Figures and Tables

**Table 1:** Hydrogen-bond geometry (Å, °)

*D*—H⋯*A*	*D*—H	H⋯*A*	*D*⋯*A*	*D*—H⋯*A*
N1—H1*A*⋯O3	0.86	2.13	2.972 (3)	168
N2—H2*A*⋯O2^i^	0.86	2.28	3.061 (2)	152

Symmetry code: (i) 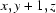.

**Fig. 1.**
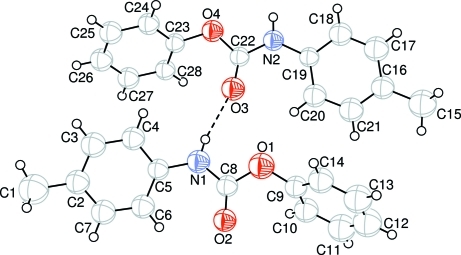
The molecular structure of the title molecule, with the atom-numbering scheme. Hydrogen bond is shown as dashed line.

**Fig. 2.**
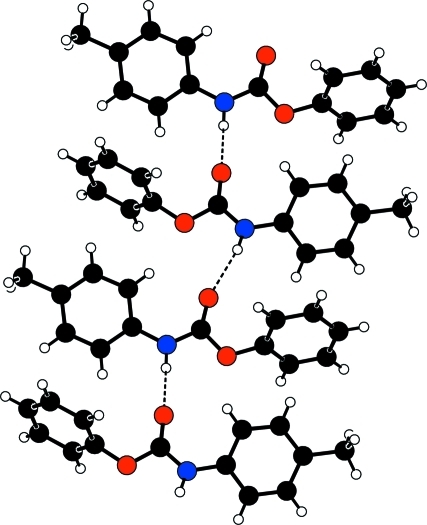
A partial packing diagram of the title compound. Hydrogen bonds are shown as dashed lines.

## supplementary crystallographic information

### Comment

Some derivatives of benzoic acid are important chemical materials. We report herein the crystal structure of the title compound.

The asymmetric unit of the title compound contains two crystallographically independent molecules (Fig. 1), in which the bond lengths (Allen *et al.*, 1987) and angles are within normal ranges. Rings A (C2-C7), B (C9-C14) and C (C16-C21), D (C23-C28) are, of course, planar and the dihedral angles between them are A/B = 59.01 (3)° and C/D = 56.98 (3)°. Intramolecular N-H···O hydrogen bond (Table 1) links the two molecules (Fig. 1).

In the crystal structure, intermolecular N-H···O hydrogen bonds (Table 1) link the molecules into chains (Fig. 2), in which they may be effective in the stabilization of the structure.

### Experimental

For the preparation of the title compound, to a cold stirring solution of *p*-toluidine (1.0 g) and triethylamine (0.8 ml) in methylene chloride (10 ml) was added phenyl chloroformate (1.0 ml) slowly keeping the temperature at 273 K. The mixture was then warmed and stirred for 1 h at room temperature. The mixture was washed with water (20 ml), dried over sodium sulfate, and concentrated to near dryness. The crude product was purified by recrystallization from petroleum ether (yield; 1.3 g). Crystals suitable for X-ray analysis were obtained by slow evaporation of a petroleum ether solution.

### Refinement

H atoms were positioned geometrically, with N-H = 0.86 Å (for NH) and C-H = 0.93 and 0.96 Å for aromatic and methyl H, respectively, and constrained to ride on their parent atoms, with U_iso_(H) = xU_eq_(C,N), where x = 1.5 for methyl H and x = 1.2 for all other H atoms.

### Crystal data

**Table:**

C_14_H_13_NO_2_	*Z* = 4
*M**_r_* = 227.25	*F*(000) = 480
Triclinic, *P*1	*D*_x_ = 1.239 Mg m^−^^3^
Hall symbol: -P 1	Mo *K*α radiation, λ = 0.71073 Å
*a* = 8.7790 (18) Å	Cell parameters from 25 reflections
*b* = 9.7470 (19) Å	θ = 9–13°
*c* = 15.121 (3) Å	µ = 0.08 mm^−^^1^
α = 87.30 (3)°	*T* = 294 K
β = 77.07 (3)°	Block, colorless
γ = 75.00 (3)°	0.30 × 0.20 × 0.10 mm
*V* = 1218.0 (5) Å^3^	

### Data collection

**Table:**

Enraf–Nonius CAD-4 diffractometer	2781 reflections with *I* > 2σ(*I*)
Radiation source: fine-focus sealed tube	*R*_int_ = 0.027
graphite	θ_max_ = 25.3°, θ_min_ = 1.4°
ω/2θ scans	*h* = 0→10
Absorption correction: ψ scan (North *et al.*, 1968)	*k* = −11→11
*T*_min_ = 0.975, *T*_max_ = 0.992	*l* = −17→18
4736 measured reflections	3 standard reflections every 120 min
4421 independent reflections	intensity decay: 1%

### Refinement

**Table:**

Refinement on *F*^2^	Secondary atom site location: difference Fourier map
Least-squares matrix: full	Hydrogen site location: inferred from neighbouring sites
*R*[*F*^2^ > 2σ(*F*^2^)] = 0.054	H-atom parameters constrained
*wR*(*F*^2^) = 0.172	*w* = 1/[σ^2^(*F*_o_^2^) + (0.097*P*)^2^] where *P* = (*F*_o_^2^ + 2*F*_c_^2^)/3
*S* = 1.01	(Δ/σ)_max_ < 0.001
4421 reflections	Δρ_max_ = 0.22 e Å^−^^3^
308 parameters	Δρ_min_ = −0.13 e Å^−^^3^
0 restraints	Extinction correction: *SHELXL97* (Sheldrick, 2008)
Primary atom site location: structure-invariant direct methods	Extinction coefficient: 0.040 (5)

### Special details

**Table:**

Geometry. All e.s.d.'s (except the e.s.d. in the dihedral angle between two l.s. planes) are estimated using the full covariance matrix. The cell e.s.d.'s are taken into account individually in the estimation of e.s.d.'s in distances, angles and torsion angles; correlations between e.s.d.'s in cell parameters are only used when they are defined by crystal symmetry. An approximate (isotropic) treatment of cell e.s.d.'s is used for estimating e.s.d.'s involving l.s. planes.
Refinement. Refinement of *F*^2^ against ALL reflections. The weighted *R*-factor *wR* and goodness of fit *S* are based on *F*^2^, conventional *R*-factors *R* are based on *F*, with *F* set to zero for negative *F*^2^. The threshold expression of *F*^2^ > σ(*F*^2^) is used only for calculating *R*-factors(gt) *etc*. and is not relevant to the choice of reflections for refinement. *R*-factors based on *F*^2^ are statistically about twice as large as those based on *F*, and *R*- factors based on ALL data will be even larger.

### Fractional atomic coordinates and isotropic or equivalent isotropic displacement parameters (Å^2^)

**Table:**

	*x*	*y*	*z*	*U*_iso_*/*U*_eq_	
O1	0.2677 (3)	0.48473 (18)	0.79602 (12)	0.0801 (6)	
O2	0.2281 (2)	0.27924 (16)	0.86169 (11)	0.0605 (5)	
O3	0.2572 (2)	0.76205 (16)	0.89540 (11)	0.0675 (5)	
O4	0.2965 (2)	0.94932 (16)	0.96266 (10)	0.0593 (5)	
N1	0.2453 (3)	0.4652 (2)	0.94342 (13)	0.0641 (6)	
H1A	0.2456	0.5534	0.9383	0.077*	
N2	0.2903 (2)	0.96349 (19)	0.81800 (12)	0.0537 (5)	
H2A	0.3101	1.0436	0.8255	0.064*	
C1	0.2787 (4)	0.2204 (4)	1.2922 (2)	0.1085 (12)	
H1B	0.3382	0.2673	1.3213	0.163*	
H1C	0.3329	0.1212	1.2845	0.163*	
H1D	0.1718	0.2311	1.3290	0.163*	
C2	0.2677 (4)	0.2855 (3)	1.20065 (18)	0.0701 (8)	
C3	0.3342 (3)	0.3980 (3)	1.16953 (19)	0.0750 (8)	
H3A	0.3871	0.4341	1.2061	0.090*	
C4	0.3240 (3)	0.4578 (3)	1.08582 (17)	0.0649 (7)	
H4A	0.3684	0.5340	1.0670	0.078*	
C5	0.2476 (3)	0.4040 (2)	1.02999 (16)	0.0543 (6)	
C6	0.1754 (3)	0.2940 (3)	1.06131 (17)	0.0677 (7)	
H6A	0.1191	0.2597	1.0259	0.081*	
C7	0.1882 (4)	0.2367 (3)	1.14483 (18)	0.0728 (8)	
H7A	0.1413	0.1622	1.1645	0.087*	
C8	0.2428 (3)	0.3982 (2)	0.86860 (16)	0.0552 (6)	
C9	0.2452 (4)	0.4455 (2)	0.71360 (17)	0.0592 (7)	
C10	0.0941 (4)	0.4490 (3)	0.7028 (2)	0.0736 (8)	
H10A	0.0059	0.4701	0.7518	0.088*	
C11	0.0741 (5)	0.4207 (3)	0.6182 (3)	0.0944 (10)	
H11A	−0.0279	0.4216	0.6098	0.113*	
C12	0.2045 (6)	0.3912 (3)	0.5469 (2)	0.0998 (12)	
H12A	0.1902	0.3734	0.4899	0.120*	
C13	0.3554 (5)	0.3876 (3)	0.5580 (2)	0.0927 (10)	
H13A	0.4439	0.3659	0.5092	0.111*	
C14	0.3751 (4)	0.4166 (3)	0.6428 (2)	0.0744 (8)	
H14A	0.4769	0.4164	0.6513	0.089*	
C15	0.2199 (4)	0.8740 (3)	0.45921 (18)	0.0983 (11)	
H15A	0.1840	0.7895	0.4568	0.148*	
H15B	0.3217	0.8649	0.4171	0.148*	
H15C	0.1416	0.9544	0.4434	0.148*	
C16	0.2396 (3)	0.8946 (3)	0.55416 (17)	0.0683 (8)	
C17	0.2895 (4)	1.0104 (3)	0.57645 (18)	0.0785 (9)	
H17A	0.3120	1.0758	0.5318	0.094*	
C18	0.3062 (3)	1.0305 (3)	0.66276 (17)	0.0681 (8)	
H18A	0.3396	1.1090	0.6756	0.082*	
C19	0.2739 (3)	0.9354 (2)	0.73064 (15)	0.0495 (6)	
C20	0.2257 (4)	0.8183 (3)	0.70921 (17)	0.0675 (7)	
H20A	0.2045	0.7521	0.7535	0.081*	
C21	0.2095 (4)	0.8008 (3)	0.62212 (18)	0.0751 (8)	
H21A	0.1769	0.7220	0.6090	0.090*	
C22	0.2787 (3)	0.8796 (2)	0.89110 (15)	0.0483 (6)	
C23	0.2708 (3)	0.8880 (2)	1.04888 (15)	0.0481 (6)	
C24	0.3933 (3)	0.8655 (3)	1.09414 (17)	0.0587 (7)	
H24A	0.4928	0.8812	1.0660	0.070*	
C25	0.3663 (3)	0.8190 (3)	1.18239 (17)	0.0662 (7)	
H25A	0.4482	0.8031	1.2143	0.079*	
C26	0.2183 (3)	0.7959 (3)	1.22356 (17)	0.0677 (8)	
H26A	0.2004	0.7656	1.2834	0.081*	
C27	0.0979 (3)	0.8173 (3)	1.17687 (17)	0.0632 (7)	
H27A	−0.0010	0.7998	1.2046	0.076*	
C28	0.1224 (3)	0.8648 (2)	1.08853 (16)	0.0543 (6)	
H28A	0.0405	0.8808	1.0566	0.065*	

### Atomic displacement parameters (Å^2^)

**Table:**

	*U*^11^	*U*^22^	*U*^33^	*U*^12^	*U*^13^	*U*^23^
O1	0.1460 (19)	0.0512 (11)	0.0592 (11)	−0.0492 (12)	−0.0287 (11)	0.0079 (9)
O2	0.0878 (13)	0.0380 (9)	0.0662 (11)	−0.0274 (8)	−0.0262 (9)	0.0036 (8)
O3	0.1163 (15)	0.0379 (9)	0.0578 (10)	−0.0340 (9)	−0.0233 (10)	0.0068 (8)
O4	0.0911 (13)	0.0498 (9)	0.0516 (10)	−0.0397 (9)	−0.0221 (9)	0.0072 (8)
N1	0.1033 (17)	0.0368 (10)	0.0604 (13)	−0.0304 (11)	−0.0202 (12)	0.0007 (9)
N2	0.0792 (14)	0.0374 (10)	0.0520 (11)	−0.0288 (10)	−0.0149 (10)	0.0062 (9)
C1	0.124 (3)	0.111 (3)	0.068 (2)	0.001 (2)	−0.014 (2)	0.0120 (19)
C2	0.0782 (19)	0.0606 (17)	0.0556 (16)	0.0035 (15)	−0.0058 (14)	−0.0036 (13)
C3	0.0718 (19)	0.092 (2)	0.0628 (17)	−0.0202 (16)	−0.0164 (14)	−0.0093 (16)
C4	0.0734 (18)	0.0625 (17)	0.0647 (17)	−0.0305 (14)	−0.0102 (14)	−0.0079 (13)
C5	0.0695 (16)	0.0394 (13)	0.0518 (14)	−0.0140 (11)	−0.0081 (12)	−0.0026 (11)
C6	0.098 (2)	0.0527 (15)	0.0593 (16)	−0.0330 (15)	−0.0154 (15)	−0.0012 (12)
C7	0.104 (2)	0.0464 (15)	0.0622 (17)	−0.0221 (15)	−0.0031 (16)	0.0012 (13)
C8	0.0731 (17)	0.0375 (13)	0.0597 (15)	−0.0221 (12)	−0.0157 (13)	0.0058 (11)
C9	0.085 (2)	0.0373 (13)	0.0572 (16)	−0.0205 (13)	−0.0161 (14)	0.0082 (11)
C10	0.083 (2)	0.0533 (16)	0.082 (2)	−0.0169 (15)	−0.0160 (17)	0.0110 (14)
C11	0.116 (3)	0.078 (2)	0.108 (3)	−0.034 (2)	−0.057 (2)	0.025 (2)
C12	0.178 (4)	0.068 (2)	0.067 (2)	−0.037 (2)	−0.049 (3)	0.0109 (17)
C13	0.122 (3)	0.073 (2)	0.068 (2)	−0.020 (2)	0.003 (2)	0.0026 (16)
C14	0.082 (2)	0.0612 (17)	0.078 (2)	−0.0220 (15)	−0.0114 (17)	0.0098 (15)
C15	0.145 (3)	0.087 (2)	0.0587 (18)	−0.012 (2)	−0.0344 (19)	−0.0033 (16)
C16	0.089 (2)	0.0528 (16)	0.0532 (15)	−0.0030 (14)	−0.0119 (14)	−0.0038 (12)
C17	0.118 (3)	0.0602 (17)	0.0537 (16)	−0.0259 (17)	−0.0112 (16)	0.0120 (13)
C18	0.101 (2)	0.0509 (15)	0.0568 (16)	−0.0327 (15)	−0.0123 (14)	0.0076 (12)
C19	0.0624 (15)	0.0360 (12)	0.0486 (13)	−0.0127 (11)	−0.0089 (11)	−0.0003 (10)
C20	0.108 (2)	0.0471 (14)	0.0579 (16)	−0.0333 (15)	−0.0255 (15)	0.0080 (12)
C21	0.122 (3)	0.0504 (15)	0.0629 (17)	−0.0303 (16)	−0.0309 (16)	0.0019 (13)
C22	0.0600 (15)	0.0367 (12)	0.0510 (13)	−0.0179 (11)	−0.0113 (11)	−0.0010 (10)
C23	0.0665 (16)	0.0354 (12)	0.0480 (13)	−0.0194 (11)	−0.0163 (12)	−0.0001 (10)
C24	0.0585 (16)	0.0569 (15)	0.0679 (17)	−0.0249 (12)	−0.0173 (13)	0.0042 (12)
C25	0.0712 (18)	0.0742 (18)	0.0652 (17)	−0.0273 (15)	−0.0311 (14)	0.0094 (14)
C26	0.089 (2)	0.0717 (18)	0.0483 (15)	−0.0304 (16)	−0.0177 (14)	0.0076 (13)
C27	0.0618 (17)	0.0673 (17)	0.0601 (16)	−0.0225 (13)	−0.0063 (13)	0.0052 (13)
C28	0.0555 (15)	0.0538 (14)	0.0587 (15)	−0.0194 (12)	−0.0176 (12)	0.0039 (11)

### Geometric parameters (Å, °)

**Table:**

O1—C8	1.366 (3)	C11—H11A	0.9300
O1—C9	1.390 (3)	C12—C13	1.363 (5)
O2—C8	1.211 (3)	C12—H12A	0.9300
O3—C22	1.205 (3)	C13—C14	1.384 (4)
O4—C22	1.363 (3)	C13—H13A	0.9300
O4—C23	1.404 (3)	C14—H14A	0.9300
N1—C5	1.415 (3)	C15—C16	1.512 (3)
N1—C8	1.340 (3)	C15—H15A	0.9600
N1—H1A	0.8600	C15—H15B	0.9600
N2—C19	1.407 (3)	C15—H15C	0.9600
N2—C22	1.345 (3)	C16—C21	1.370 (4)
N2—H2A	0.8600	C16—C17	1.392 (4)
C1—C2	1.508 (4)	C17—C18	1.374 (4)
C1—H1B	0.9600	C17—H17A	0.9300
C1—H1C	0.9600	C18—C19	1.381 (3)
C1—H1D	0.9600	C18—H18A	0.9300
C2—C7	1.378 (4)	C19—C20	1.390 (3)
C2—C3	1.387 (4)	C20—C21	1.379 (3)
C3—C4	1.380 (4)	C20—H20A	0.9300
C3—H3A	0.9300	C21—H21A	0.9300
C4—C5	1.383 (3)	C23—C24	1.366 (3)
C4—H4A	0.9300	C23—C28	1.377 (3)
C5—C6	1.395 (3)	C24—C25	1.378 (3)
C6—C7	1.372 (3)	C24—H24A	0.9300
C6—H6A	0.9300	C25—C26	1.380 (4)
C7—H7A	0.9300	C25—H25A	0.9300
C9—C14	1.357 (4)	C26—C27	1.366 (3)
C9—C10	1.364 (4)	C26—H26A	0.9300
C10—C11	1.379 (4)	C27—C28	1.382 (3)
C10—H10A	0.9300	C27—H27A	0.9300
C11—C12	1.365 (5)	C28—H28A	0.9300
			
C8—O1—C9	118.14 (18)	C14—C13—H13A	120.5
C22—O4—C23	118.29 (16)	C9—C14—C13	119.7 (3)
C5—N1—H1A	117.0	C9—C14—H14A	120.2
C8—N1—C5	125.91 (19)	C13—C14—H14A	120.2
C8—N1—H1A	117.0	C16—C15—H15A	109.5
C19—N2—H2A	116.2	C16—C15—H15B	109.5
C22—N2—C19	127.53 (18)	H15A—C15—H15B	109.5
C22—N2—H2A	116.2	C16—C15—H15C	109.5
C2—C1—H1B	109.5	H15A—C15—H15C	109.5
C2—C1—H1C	109.5	H15B—C15—H15C	109.5
H1B—C1—H1C	109.5	C21—C16—C17	116.9 (2)
C2—C1—H1D	109.5	C21—C16—C15	121.9 (3)
H1B—C1—H1D	109.5	C17—C16—C15	121.3 (3)
H1C—C1—H1D	109.5	C18—C17—C16	121.6 (2)
C7—C2—C3	117.1 (3)	C18—C17—H17A	119.2
C7—C2—C1	121.0 (3)	C16—C17—H17A	119.2
C3—C2—C1	121.9 (3)	C17—C18—C19	120.8 (2)
C4—C3—C2	121.9 (3)	C17—C18—H18A	119.6
C4—C3—H3A	119.1	C19—C18—H18A	119.6
C2—C3—H3A	119.1	C18—C19—C20	118.4 (2)
C3—C4—C5	119.8 (2)	C18—C19—N2	118.0 (2)
C3—C4—H4A	120.1	C20—C19—N2	123.7 (2)
C5—C4—H4A	120.1	C21—C20—C19	119.8 (2)
C4—C5—C6	119.1 (2)	C21—C20—H20A	120.1
C4—C5—N1	117.9 (2)	C19—C20—H20A	120.1
C6—C5—N1	123.0 (2)	C16—C21—C20	122.7 (3)
C7—C6—C5	119.5 (3)	C16—C21—H21A	118.7
C7—C6—H6A	120.3	C20—C21—H21A	118.7
C5—C6—H6A	120.3	O3—C22—N2	127.6 (2)
C6—C7—C2	122.5 (3)	O3—C22—O4	124.0 (2)
C6—C7—H7A	118.8	N2—C22—O4	108.47 (18)
C2—C7—H7A	118.8	C24—C23—C28	122.0 (2)
O2—C8—N1	128.3 (2)	C24—C23—O4	117.1 (2)
O2—C8—O1	123.1 (2)	C28—C23—O4	120.6 (2)
N1—C8—O1	108.56 (19)	C23—C24—C25	118.6 (2)
C14—C9—C10	121.4 (3)	C23—C24—H24A	120.7
C14—C9—O1	117.9 (3)	C25—C24—H24A	120.7
C10—C9—O1	120.4 (3)	C24—C25—C26	120.3 (2)
C9—C10—C11	119.0 (3)	C24—C25—H25A	119.9
C9—C10—H10A	120.5	C26—C25—H25A	119.9
C11—C10—H10A	120.5	C27—C26—C25	120.3 (2)
C12—C11—C10	119.8 (3)	C27—C26—H26A	119.9
C12—C11—H11A	120.1	C25—C26—H26A	119.9
C10—C11—H11A	120.1	C26—C27—C28	120.2 (2)
C13—C12—C11	121.1 (3)	C26—C27—H27A	119.9
C13—C12—H12A	119.5	C28—C27—H27A	119.9
C11—C12—H12A	119.5	C23—C28—C27	118.5 (2)
C12—C13—C14	119.0 (3)	C23—C28—H28A	120.7
C12—C13—H13A	120.5	C27—C28—H28A	120.7
			
C7—C2—C3—C4	−1.0 (4)	C21—C16—C17—C18	−0.7 (4)
C1—C2—C3—C4	−179.9 (3)	C15—C16—C17—C18	179.4 (3)
C2—C3—C4—C5	−0.9 (4)	C16—C17—C18—C19	0.1 (5)
C3—C4—C5—C6	2.9 (4)	C17—C18—C19—C20	0.6 (4)
C3—C4—C5—N1	−177.7 (2)	C17—C18—C19—N2	−178.8 (2)
C8—N1—C5—C4	149.9 (3)	C22—N2—C19—C18	−173.8 (2)
C8—N1—C5—C6	−30.8 (4)	C22—N2—C19—C20	6.8 (4)
C4—C5—C6—C7	−3.1 (4)	C18—C19—C20—C21	−0.8 (4)
N1—C5—C6—C7	177.6 (2)	N2—C19—C20—C21	178.6 (3)
C5—C6—C7—C2	1.2 (4)	C17—C16—C21—C20	0.5 (4)
C3—C2—C7—C6	0.8 (4)	C15—C16—C21—C20	−179.5 (3)
C1—C2—C7—C6	179.7 (3)	C19—C20—C21—C16	0.2 (5)
C5—N1—C8—O2	6.9 (4)	C19—N2—C22—O3	2.7 (4)
C5—N1—C8—O1	−170.2 (2)	C19—N2—C22—O4	−177.8 (2)
C9—O1—C8—O2	12.4 (4)	C23—O4—C22—O3	−7.4 (3)
C9—O1—C8—N1	−170.3 (2)	C23—O4—C22—N2	173.05 (18)
C8—O1—C9—C14	−116.7 (3)	C22—O4—C23—C24	124.8 (2)
C8—O1—C9—C10	69.1 (3)	C22—O4—C23—C28	−61.1 (3)
C14—C9—C10—C11	0.8 (4)	C28—C23—C24—C25	−0.4 (4)
O1—C9—C10—C11	174.8 (2)	O4—C23—C24—C25	173.5 (2)
C9—C10—C11—C12	−0.7 (4)	C23—C24—C25—C26	0.1 (4)
C10—C11—C12—C13	0.8 (5)	C24—C25—C26—C27	0.8 (4)
C11—C12—C13—C14	−1.1 (5)	C25—C26—C27—C28	−1.2 (4)
C10—C9—C14—C13	−1.1 (4)	C24—C23—C28—C27	0.0 (4)
O1—C9—C14—C13	−175.2 (2)	O4—C23—C28—C27	−173.8 (2)
C12—C13—C14—C9	1.2 (4)	C26—C27—C28—C23	0.8 (4)

### Hydrogen-bond geometry (Å, °)

**Table:**

*D*—H···*A*	*D*—H	H···*A*	*D*···*A*	*D*—H···*A*
N1—H1A···O3	0.86	2.13	2.972 (3)	168
N2—H2A···O2^i^	0.86	2.28	3.061 (2)	152

Symmetry codes: (i) *x*, *y*+1, *z*.
